# Effectiveness of an impedance cardiography guided treatment strategy to improve blood pressure control in a real-world setting: results from a pragmatic clinical trial

**DOI:** 10.1136/openhrt-2021-001719

**Published:** 2021-09-27

**Authors:** Luyan Wang, Yuan Lu, Hongyi Wang, Jianlei Gu, Zheng J Ma, Zheng Lian, Zhiying Zhang, Harlan Krumholz, Ningling Sun

**Affiliations:** 1 Department of Cardiology and Hypertension, Peking University People's Hospital, Beijing, China; 2 Section of Cardiovascular Medicine, Department of Internal Medicine, Yale School of Medicine, New Haven, Connecticut, USA; 3 Center for Outcomes Research and Evaluation, Yale-New Haven Hospital, New Haven, Connecticut, USA; 4 SJTU-Yale Joint Center for Biostatistics, Shanghai Jiao Tong University, Shanghai, China; 5 Shanghai Engineering Research Center for Big Data in Pediatric Precision Medicine, Shanghai, China; 6 Department of Research & Development, Beijing Li-Heng Medical Technologies, Ltd, Beijing, China; 7 Department of Cardiology, Beijing Tiantan Puhua Hospital, Beijing, China; 8 Department of Health Policy and Management, Yale School of Medicine, New Haven, Connecticut, USA

**Keywords:** hypertension, epidemiology, delivery of health care

## Abstract

**Objective:**

To test the effectiveness of an impedance cardiography (ICG) guided treatment strategy on improving blood pressure (BP) control in real-world clinical practice.

**Design:**

A single-centre, pragmatic randomised trial.

**Setting:**

A hypertension clinic of the Peking University People’s Hospital in Beijing, China.

**Participants:**

Adults who sought outpatient care for hypertension in the hypertension clinic at the Peking University People’s Hospital between June and December 2019.

**Interventions:**

A computerised clinical decision support of recommending treatment choices to providers based on patients’ haemodynamic profiles measured by ICG.

**Main outcome measures:**

Changes in systolic BP (SBP) and diastolic BP (DBP) levels at the follow-up visit 4–12 weeks after baseline. Secondary outcomes included achievement of BP goal of <140/90 mm Hg and the changes in BP by baseline BP, age, sex and body mass index (BMI).

**Results:**

A total of 102 adults (mean age was 54±14 years; 41% were women) completed the study. The mean baseline SBP was 150.9 (SD of 11.5) mm Hg and mean baseline DBP was 91.1 (11.3) mm Hg. At the follow-up visit, the mean SBP and DBP decreased by 19.9 and 11.3 mm Hg in the haemodynamic group, as compared with 12.0 and 4.9 mm Hg in the standard care group (p value for difference between groups <0.001). The proportion of patients achieving BP goal of <140/90 mm Hg in the haemodynamic group was 67%, as compared with 41% in the standard care group (p=0.017). The haemodynamic group had a larger effect on BP reduction consistently across subgroups by age, sex, BMI and baseline BP.

**Conclusions:**

An ICG-guided treatment strategy led to greater reductions in BP levels than were observed with standard care in a real-world population of outpatients with hypertension. There is a need for further validation of this strategy for improving blood pressure treatment selection.

**Trial registration number:**

NCT04715698.

**Key questions**

**What is already known about this subject?**

- Hypertension is a hemodynamic-related disorder characterized by abnormalities of the cardiac output, systemic vascular resistance, or a combination of both. Measurement of the various hemodynamic parameters using impedance cardiography (ICG) in stable patients with hypertension provides information that may enable more effective targeted drug management.

**What does this study add?**

- This study shows an ICG-guided treatment strategy could lead to greater reductions in blood pressure levels than were observed with standard care in a real-world population of outpatients with hypertension.

**How might this impact on clinical practice?**

- As clinical care is moving towards precision medicine, our findings identify the needs of more refined hemodynamic measurement to facilitate personalized treatment in patients with hypertension.

## Background

Hypertension is a haemodynamic-related disorder characterised by abnormalities of the cardiac output (CO), systemic vascular resistance (SVR) or a combination of both.^1^ Despite that hypertension is routinely diagnosed and managed based on degree of blood pressure (BP) elevation alone, patients with similar degree of BP elevation can have different underlying haemodynamic profiles.^2 3^ These variations in haemodynamic profiles may have important implications for treatment selection because the choice for patients with a higher CO might be different than for those with a higher SVR. Selecting treatment strategies based on haemodynamic profiles for patients with hypertension may improve BP control.

Impedance cardiography (ICG) is a safe and accurate non-invasive tool to measure haemodynamic parameters^4 5^ that can be performed in the outpatient setting.^6 7^ Measurement of the various haemodynamic components using ICG in stable patients with hypertension provides information that may enable more effective targeted drug management. Although several previous studies have used ICG to evaluate haemodynamic parameters and demonstrated that ICG-guided therapy improves BP control,^7–9^ they used a traditional randomised controlled trial design, in which the operationalisation of the intervention had stricter instructions and patients were more frequently monitored than routine clinical care. Whether an ICG-guided strategy for hypertension treatment can lead to improvements in BP control in real-world clinical settings has been rarely tested. Additionally, previous studies were all conducted in the USA^8 9^; no study has focused on low-income and middle-income counties where healthcare resources are limited, patient characteristics and clinical practice patterns are different.

Accordingly, we conducted a pragmatic randomised trial to produce preliminary data about the effectiveness of ICG-guided strategies for patients with hypertension in routine clinical care in China. We hypothesised that selecting antihypertensive therapy based on each patient’s haemodynamic profile measured by ICG could lead to more effective BP reduction and hypertension control than standard care in hypertensive patients in a real-world setting.

## Methods

### Eligibility

The study population was patients who sought outpatient care for hypertension in the hypertension clinic of the Cardiology Department at the Peking University People’s Hospital between June and December 2019 in Beijing, China. Patients were eligible if they were 18–85 years old, were local residents, had a diagnosis of essential hypertension and were currently on less than four antihypertensive medications of different classes with systolic BP (SBP) of ≥140 mm Hg or diastolic BP (DBP) of ≥90 mm Hg. If patients were on a combination antihypertensive drug, they would be considered on multiple classes of antihypertensive drugs. Patients were excluded if they were already on four or more antihypertensive agents of different classes (considered as resistant hypertension); had on-site SBP of <140 mm Hg and DBP of <90 mm Hg; had secondary hypertension, severe renal disease, cancer, severe valvular disease, cerebrovascular event within 6 months, atrial fibrillation; or had uncontrolled diabetes with fasting blood glucose of 11.1 mmol/L.

### Randomisation and procedure

After informed consent, patients meeting inclusion/exclusion criteria were randomised in a 1:1 ratio to the haemodynamic group or the standard care group. Simple randomisation was performed using a random number generator with concealed allocation. Randomisation was performed at the patient rather than the provider level, as outpatients at the participating clinic may be cared for by different providers throughout the study. All study investigators were blinded to patient randomisation status until enrolment was complete.

Patients’ information including age, sex, weight, height, BP and antihypertensive medications was collected by nurses during the outpatient visit. Weight was measured to the nearest 0.1 kg with patients wearing light indoor clothing and no shoes. Height was measured to the nearest 0.1 cm, using a portable stadiometer (Omron HNJ-318; Omron Corporation, Kyoto, Japan) with patients standing without shoes and heels against the wall. BP was measured on the right upper arm after 5 min of rest in a seated position using an electronic BP monitor (Omron HBP-9020; Omron Corporation). ICG data were collected by trained technicians at each visit in all patients, but ICG findings were not revealed in the standard arm to physicians or patients. ICG was performed with patients in the supine position, resting for 3 min before measurement. By applying a constant, low amplitude, high-frequency, alternating electrical current to the thorax, ICG device measures the corresponding voltage to detect beat-to-beat changes in thoracic electrical resistance, known as impedance and with it stroke volume is estimated.^10 11^ Then, using heart rate, mean arterial BP and BMI, other haemodynamic parameters are calculated, including CO, cardiac index (CI), SVR, SVR index (SVRI), arterial stiffness index (AS) and a volume parameter—thoracic blood saturation ratio (TBR).^12^ The ICG device used (CHM P2505, designed by Beijing Li-Heng Medical Technologies, manufactured by Shandong Baolihao Medical Appliances) was developed based on improved hardware and advanced digital filtering algorithms,^13^ and has been validated versus both invasive thermodilution and non-invasive echocardiography in different settings.^14–16^

### Intervention

**Figure 1:**
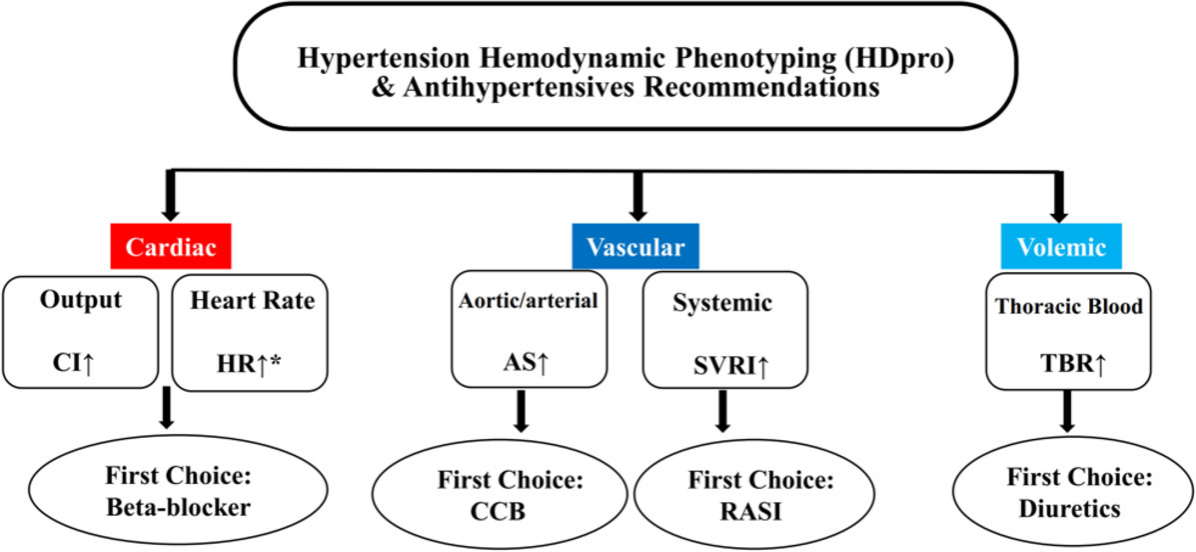
Suggested treatment strategy for the haemodynamic group. CCB, calcium channel blockers; RASI, renin-angiotensin system inhibitors. In case of evaluation of multiple indicators, first consider the highest one, or considering drug combinations. In case that the recommended drug is already used, consider dose titration, switching to extended release formulation or switching to a different in the same drug class. Based on hemodynamic phenotyping, final drug choices should take in consideration comorbidity and other clinical info. ↑ : Value greater than “baseline + 1SD”, baseline and SD based on large sample Chinese general population, adjusted for individual factors such as: age, sex, height and weight. HR ↑ *: HR>75 b/min; But in case of low cardiac output (CI<baseline-2SD), use beta-blockers cautiously. AS, aortic/arterial stiffness; CI, cardiac index; SVRI, systemic vascular resistance index; TBR, thoracic blood standing/supine ratio.

After randomisation, therapy was initiated in all patients. Physicians in both groups were encouraged to prescribe medications consistent with the 2018 Chinese hypertension guideline,^17^ their clinical judgement, and patient clinical characteristics. In the haemodynamic group, physicians were provided with patients’ ICG findings and a computerised clinical decision support of recommended treatment choices based on patients’ haemodynamic profiles. Specifically, the clinical decision support system determined the haemodynamic phenotype of a patient in three steps: first, the computer system calculated the population mean and SD of each haemodynamic parameter (eg, HR, CI, AS, SVRI, TBR) given patient’s gender, age, weight, height and BMI, using data from a large sample of 114 198 generally healthy Chinese adults (see detailed description in online supplemental file 1).^2 3^ Because haemodynamic parameters vary by age, gender, height and weight, we used personalised cutoffs as opposed to one-size-fits-all cutoffs to define haemodynamic phenotypes. Second, the computer system determined if the patient had an elevated haemodynamic parameter based on whether the patient’s value was greater than the population mean plus one SD of the respective parameter. Finally, the clinical decision support categorised patients into four clinically relevant haemodynamic phenotypes, including cardiac phenotype (high HR or high CI), arterial vascular phenotype (high AS), peripheral vascular phenotype (high SVRI) and volemic phenotype (high TBR).^18 19^ These four haemodynamic phenotypes included cardiac phenotype (high HR or high CI), arterial vascular phenotype (high AS), peripheral vascular phenotype (high SVRI) and volemic phenotype (high TBR). Suggested treatment strategies were then provided for each phenotype (see details in figure 1). Physicians were instructed to use this information to guide decisions about pharmacological agents and dosing. Physicians could share ICG information with patients in the haemodynamic arm. In the standard care group, physicians were not provided with patients’ ICG findings and were instructed to use their own clinical judgement to make treatment decisions. To minimise the potential confounding due to lifestyle modification, physicians in both groups were instructed not to prescribe non-pharmacological interventions as part of their treatment plans. All patients in both groups received education on the importance of medication compliance.

10.1136/openhrt-2021-001719.supp1 Supplementary data

### Outcome measures

All patients were required to return to the clinic for a follow-up visit between 4 and 12 weeks after the baseline visit. During the follow-up visit, BP was measured on the right upper arm after 5 min of rest in a seated position using an electronic BP monitor (Omron HBP-9020). The technicians who measured BP were blinded to the intervention arm. The primary study end points were changes in SBP and DBP from baseline. Secondary study end points included (1) achievement of BP goal of <140/90 mm Hg and (2) changes in SBP and DBP by baseline BP, age, sex and BMI.

### Statistical analysis

We described continuous variables as mean±SD and categorical variables as n (%). Differences in continuous variables between treatment groups were examined by the Student’s t-test and in categorial variables using Fisher’s exact tests. Subgroup analysis was performed by baseline BP, age, sex, BMI and haemodynamic phenotype. We used Breslow-Day test to test the consistency of different stratified OR across subgroups and used Forest plots for visualisation. We performed additional evaluation of changes in haemodynamic parameters between baseline and follow-up visit by pair-sample t-test. Statistical significance was defined as a two-tailed p<0.05. All statistical analyses were conducted using R, V.3.4.1 (The R Foundation for Statistical Computing, Vienna, Austria). The study followed the guidelines for randomised trial, described in the Consolidated Standards of Reporting Trials statement.^20^

### Patient involvement

No patients were involved in the development of the research question or the outcome measures, or in developing plans for the design and implementation of the study. The data are deidentified and, therefore, cannot be shared with the study participants directly.

## Results

### Characteristics of study population

Between 1 June and 31 December 2019, we screened 201 patients presenting to the hypertension clinic for outpatient care, from which we excluded 87 individuals whose baseline BP value is less than 140/90 mm Hg, leaving 114 patients randomised to the intervention and control arms. We further excluded 12 patients who did not make follow-up visits within 4–12 weeks. Finally, a total of 102 patients (51 in the standard care group and 51 in the haemodynamic group) completed the study and were analysed (online supplemental figure 1). Among 102 patients, the mean age of 54±14 years and 41% were female. Patients had a mean SBP of 150.9 (±11.5) mm Hg, mean DBP of 91.1 (±11.3) mm Hg, mean CI of 3.1 (±0.7) L/min/m^2^, mean SVRI of 3017 (±731) dynes s/cm^5^/m^2^, mean heart rate of 72 (±10.6) beats/min (table 1).

**Table 1:** Characteristics of study participants at baseline

	Overall (n=102)	Intervention group (n=51)	Control group (n=51)
Male, n (%)	60 (59%)	32 (63%)	28 (55%)
Female, n (%)	42 (41%)	19 (37%)	23 (45%)
Age, mean (SD)	54±14.0	55±12.4	54±15.5
BMI (kg/m^2^)	26.7±3.8	26.4±3.7	27.0±3.9
SBP (mm Hg)	150.9±11.5	151.8±12.6	150.0±10.3
DBP (mm Hg)	91.1±11.3	92.7±9.6	89.5±12.6
HR (BPM)	72±10.6	73±11.2	70.0±10.0
CI (L/min/m^2^)	3.1±0.72	3.1±0.64	3.0±0.80
AS (mm Hg/mL/b)	0.82±0.33	0.82±0.36	0.82±0.30
SVRI (dyn s m^2^/cm^5^)	3017±731	3057±678	2975±720
TBR (%)	0.78±0.11	0.79±0.11	0.76±0.10
Diabetes, n (%)	25 (25%)	13 (25%)	12 (24%)
CHD, n (%)	4 (4%)	2 (4%)	2 (4%)
Stoke, n (%)	1 (1%)	1 (2%)	0 (0%)
CKD, n (%)	9 (9%)	7 (14%)	2 (4%)

The data of the two groups were not statistically significant (p>0.05 for all).

AS, aortic resistance index; BMI, body mass index; CHD, coronary heart disease; CI, cardiac output index; CKD, chronic kidney disease; DBP, diastolic blood pressure; HR, heart rate; SBP, systolic blood pressure; SVRI, systemic vascular resistance index; TBR, thoracic blood volume saturation.

In the haemodynamic group, 13 patients had cardiac phenotype (high HR or high CI), 11 had arterial vascular phenotype (high AS), 30 had peripheral vascular phenotype (high SVRI) and 17 volemic phenotype (high TBR), respectively. In the control group, 13 patients had cardiac phenotype, 18 had arterial vascular phenotype, 26 had peripheral vascular phenotype and 11 volemic phenotype, respectively. There were no statistically significant differences in the number and class of antihypertensive medications, patient demographic, clinical, BP or ICG variables at baseline between the haemodynamic group and the control group (table 1 and online supplemental tables 1–3). Baseline ICG variables and patient characteristics by haemodynamic phenotype were presented in online supplemental tables 1 and 2.

### Effect of the ICG-guided treatment strategy on BP control

**Figure 2:**
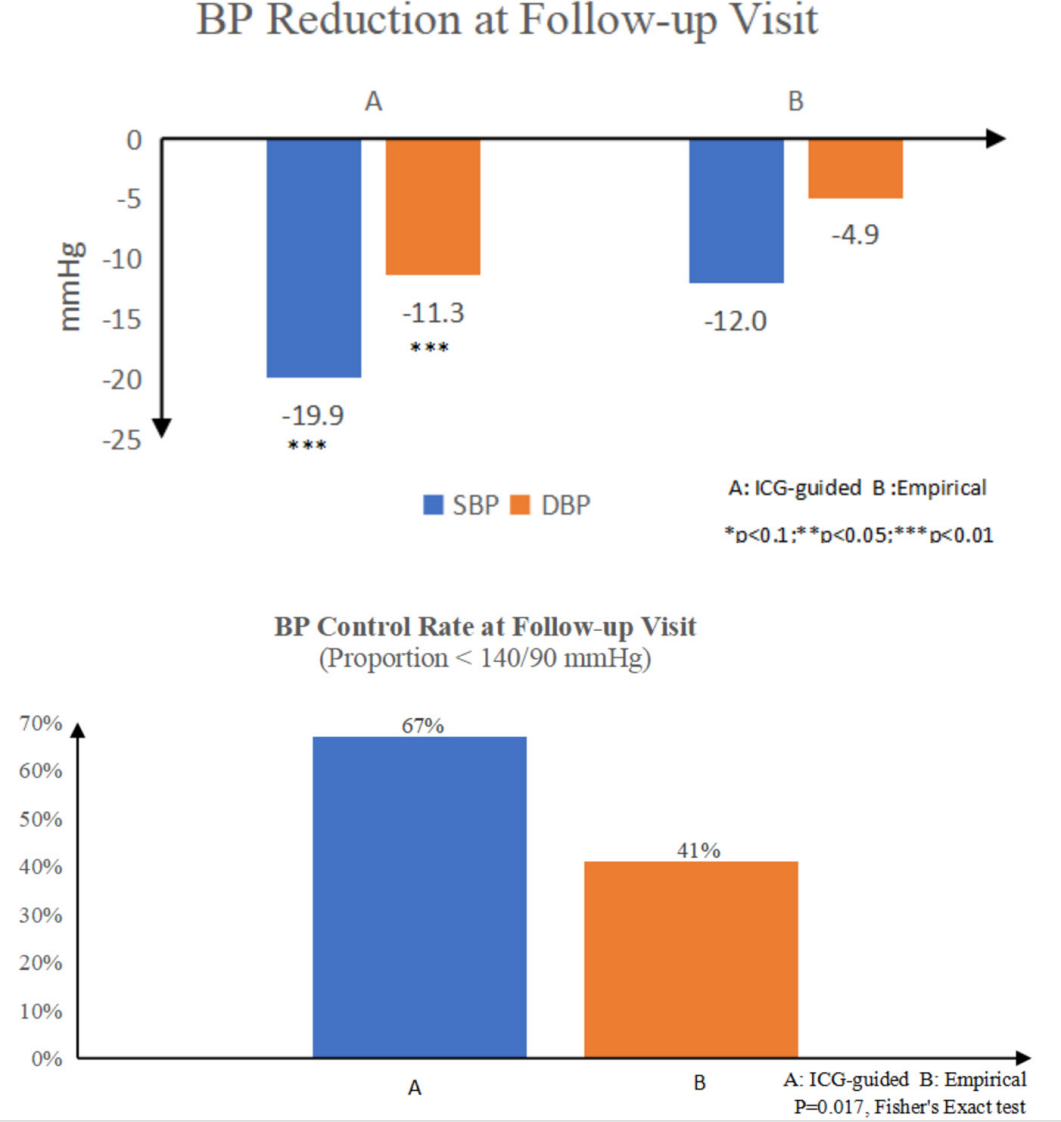
Blood pressure (BP) reduction and achievement of BP goals in haemodynamic and standard care groups. A: Haemodynamic group, B: standard care group. DBP, diastolic BP; ICG, impedance cardiography; SBP, systolic BP.

BP and ICG values at the baseline and follow-up visit as well as their differences between the two visits are shown in online supplemental table 4 and figure 2. Both SBP and DBP reductions were significantly greater in the haemodynamic group from baseline to follow-up visit compared with the standard care group (SBP reductions: 19.9±10.7 vs 12.0±11.8 mm Hg, p<0.001; DBP reduction: 11.3±6.2 vs 4.9±9.9 mm Hg, p<0.001). Final BP was lower in the haemodynamic group compared with the standard care group (SBP: 131.9±10.9 vs 138.0±13.7 mm Hg, p<0.001; DBP: 81.4±7.7 vs 84.6±12.9 mm Hg, p<0.001). The proportion of patients achieving BP goal of <140/90 mm Hg was also larger in the haemodynamic group compared with the standard care group (67% vs 41%; p=0.017).

**Figure 3:**
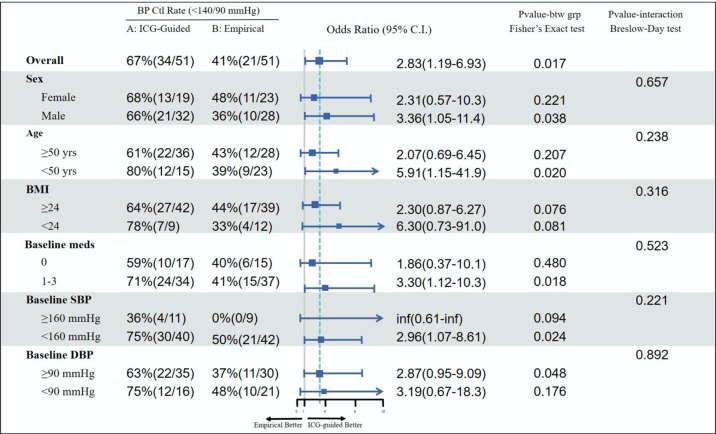
Achievement of BP goals by age, sex, BMI, baseline BP, use of medication at baseline. AS, aortic resistance index; BMI, body mass index; BP, blood pressure; DBP, diastolic BP; ICG, impedance cardiography; SBP, systolic BP; SVRI, systemic vascular resistance index.

Subgroup analyses by patient gender (men vs women), age (≥50 years vs <50 years), BMI (≥24 vs <24 kg/m2) and baseline BP level (baseline SBP ≥160 vs 140–159 mm Hg; baseline DBP ≥90 vs <90 mm Hg) have consistently shown a greater BP reduction in the haemodynamic group compared with the standard care group. The differences between the two groups were statistically significant for all subgroups, except for DBP in men, SBP in the age of <50 years and DBP in BMI of <24 kg/m^2^ where the differences between the two groups were non-significant. The proportion of patients achieving BP goal of <140/90 mm Hg was statistically significantly larger in the haemodynamic group compared with the standard care group for subgroups of men, age of <50 years, baseline SBP of <160 mm Hg and baseline DBP of ≥90 mm Hg (figure 3).

**Figure 4:**
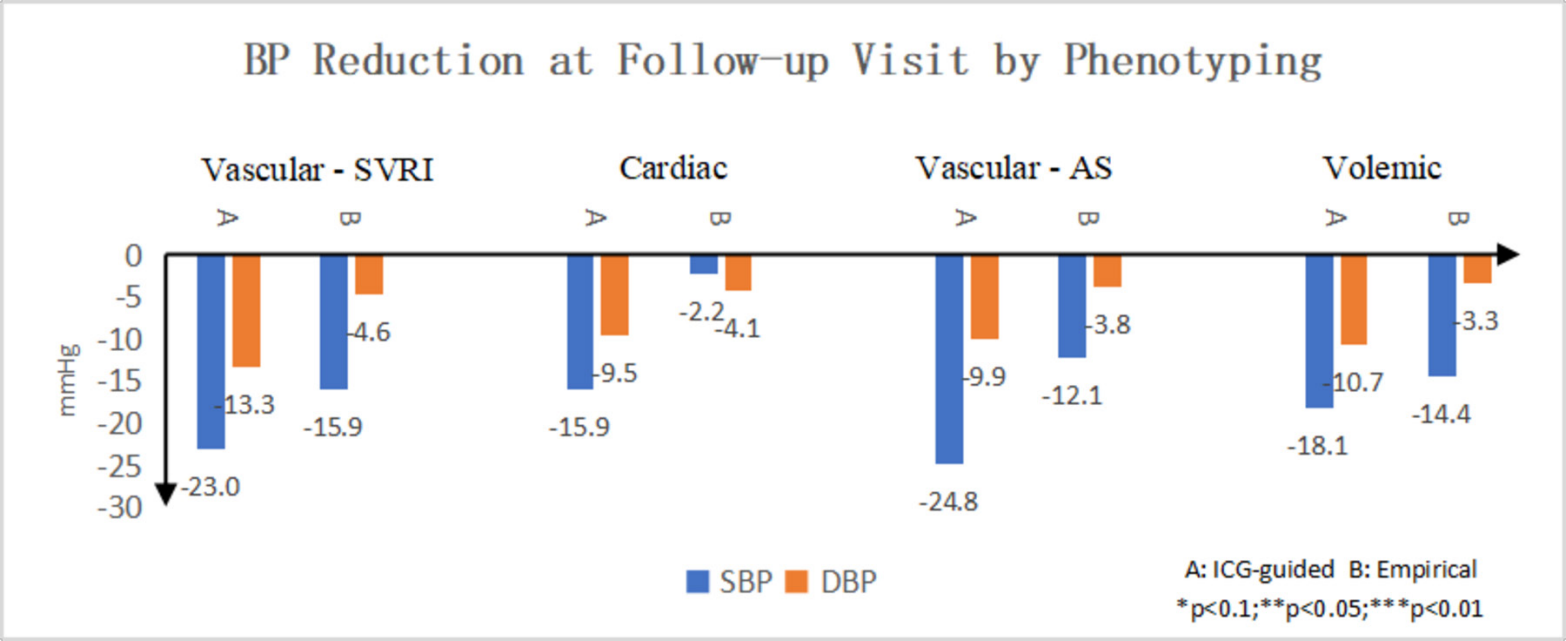
BP reduction by patients with different haemodynamic phenotypes. A: Haemodynamic group, B: standard care group. AS, arterial stiffness index; BP, blood pressure; DBP, diastolic BP; ICG, impedance cardiography; SBP, systolic BP; SVRI, systemic vascular resistance index.

Figure 4 showed BP reduction between two treatment groups by haemodynamic phenotypes. BP reduction was significantly larger in haemodynamic group compared with the standard care group for patients with hyperdynamic phenotype (high HR or high CI), arterial hyper-resistive phenotype (high AS) and peripheral artery hyper-resistive phenotype (high SVRI). BP reduction was not statistically significant in patients with high volume phenotype (high TBR).

### Correlation between antihypertensive agents and changes in haemodynamic parameters

**Figure 5:**
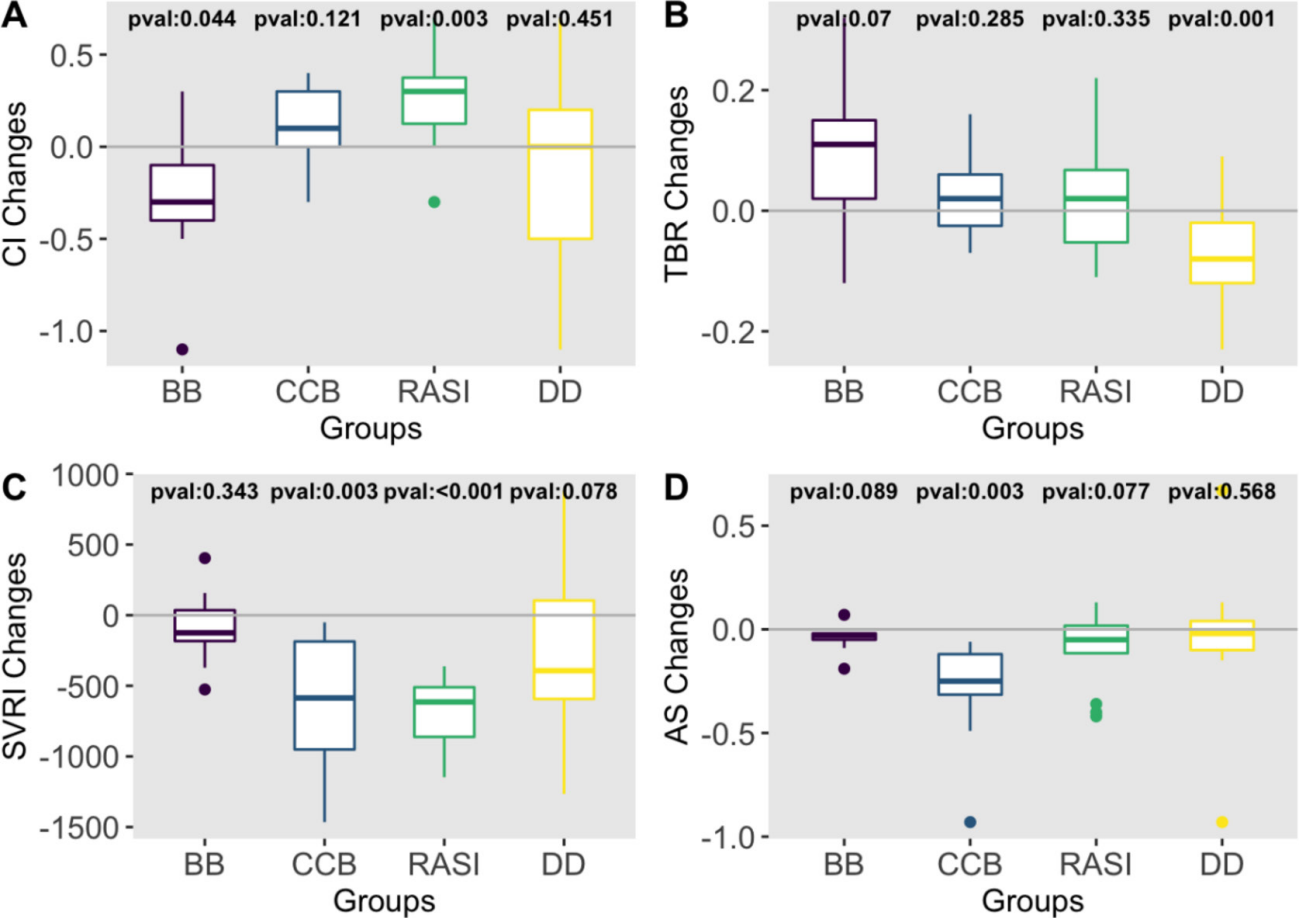
Impact of different antihypertensive agents on haemodynamic parameters. AS, arterial stiffness index; BB, beta-blockers; CB, calcium channel blockers; CI, cardiac index; DD, diuretics; RASI, renin‐angiotensin system inhibitors; TBR, thoracic blood saturation ratio.

In haemodynamic group, CI was statistically significantly reduced from baseline to follow-up visit in patients treated with beta-blockers (p=0.044, figure 5). TBR was statistically significantly reduced in patients treated with thiazide or thiazide-like diuretics (p=0.001). Both AS and SVRI were statistically significantly reduced in patients treated with calcium channel blockers (p=0.003), and SVRI was statistically significantly reduced in patients treated with renin-angiotensin system inhibitors (p<0.001).

## Discussion

In a trial of stable hypertensive patients routinely seen in clinical practice in China, we showed that an ICG-guided treatment strategy was more effective in reducing BP than standard therapy. These results were consistent across subgroups based on age, sex, BMI, baseline BP and haemodynamic phenotype. Our findings suggest that antihypertensive therapy tailored to each patient’s haemodynamic abnormality could lead to more effective antihypertensive regimens and lay the groundwork for a more definitive trial.^8 9 21 22^

Of note, the reductions in BP in both groups were large, with an almost 20 mm Hg decrease in SBP in the ICG-guided intervention group. The magnitude of the BP reduction in our study is largely consistent with previous studies conducted in the USA. Smith *et al* conducted a randomised controlled trial of 164 uncontrolled hypertensive patients on 1–3 medications.^8^ After 3 months of treatment, patients in the ICG-guided group had an average SBP reduction of 19 mm Hg compared with 12 mm Hg in the standard care group. Taler *et al* randomised 104 patients with hypertension uncontrolled on two or more drugs to a 3-month trial of ICG-guided therapy or standard therapy directed by a hypertension specialist.^9^ In this study, the mean BP reduced from 169/87 mm Hg to 139/72 mm Hg in the ICG-guided group versus from 173/91 mm Hg to 147/79 mm Hg in the control group. We further extended previous studies by using a pragmatic design to test the ICG-guided intervention in real-life clinical practice and conducting the study in a low-income and middle-income country. We also provided a clinical decision support tool in addition to the ICG report to facilitate the antihypertensive treatment selection, producing a magnitude of BP improvement in routine clinical practice similar as that in the clinical trials.

There are several potential explanations for the findings in this study. First, the presumed mechanism for improved BP control with ICG-guided intervention is primarily due to personalised antihypertensive drug selection targeted at the haemodynamic cause of elevated BP. High BP results from one or more haemodynamic abnormality, including elevated CO, SVR and blood volume.^23^ Different antihypertensive agents act on different mechanisms to reduce BP by reducing CO or SVR. For example, beta-blockers block the effects of the hormone epinephrine and make the heart to beat more slowly and with less force, which then reduce CO and lower BP. Angiotensin-converting enzyme inhibitors (ACEIs)/(angiotensin receptor blockers interfere with the body’s renin-angiotensin-aldosterone system that leads to increased sodium and urine excreted, reduced resistance in blood vessels and increased venous capacity, which then reduce SVR and lower BP. Our ICG-guided intervention provides data on the underlying cause of elevated BP and uses clinical decision support to guide clinicians in selecting antihypertensive therapies targeted at the haemodynamic abnormality associated with the elevated BP, thereby maximising the BP lowering response for the given therapeutic selection.

Second, the larger reduction in BP in the intervention group may have been, in part, a reflection of improvement in therapeutic inertia. Therapeutic inertia, which refers to the failure of clinicians to initiate or intensify treatments when the BP is not at goal, has been showed as a most common cause of uncontrolled BP in actively treated patients.^24 25^ Providing clinician access to ICG findings of patients’ haemodynamic profiles and clinical decision support tool for treatment selection may reduce therapeutic inertia in the intervention group.

Finally, the improvement of BP may, at least in part, be associated with improved communications and shared decision-making between the physician and the patient. The ICG report has served as a tool for physicians to communicate with and educate patients on the underlying haemodynamic abnormalities associated with their high BP and rationale for antihypertensive therapy selection in the intervention group. Previous studies have reported that patient–physician communication is an integral part of clinical practice and patients who understand explanations from their physicians are more likely to acknowledge health problems, modify behaviour and adhere to medications accordingly.^26–28^

Our findings have important clinical implications. Current diagnosis and management of hypertension are primary based on degree of BP elevation alone, with little attention paid to the underlying haemodynamic profile. Our study provides evidence for better identification of responders to a particular treatment regimen by profiling patients based on their haemodynamic profile using a simple, non-invasive test. As clinical care is moving towards precision medicine, our findings identify the needs of more refined haemodynamic measurement to facilitate personalised treatment in patients with hypertension. Additionally, the use of ICG-guided treatment strategy to achieve greater BP control offers a potential for better short-term use of healthcare resources. This is particularly relevant in low-income and middle-income counties where resources to improve hypertension control are limited and need to be more efficiently used. Given hypertension affects over one billion adults (30% of the global adult population) in the world,^29^ such an approach has a large potential benefit in improving hypertension control and subsequently reducing a large number of cardiovascular events.

Several limitations should be considered in the interpretation of this study. First, this is a study with limited number of participants and relatively short follow-up. We did not collect long-term follow-up data, which could have been useful to assess the long-term effect of ICG-guided treatment strategies in improving BP control. Second, our findings also warrant further study in other populations, as our study was conducted among a mostly urban, working class Chinese population and thus the results may not be generalisable to other populations. Third, we did not assess medication compliance among hypertensive patients, which may affect BP values of patients in the two arms. However, we used a pragmatic design to evaluate the effectiveness of interventions and we expect the medication compliance would be analogous to the scenarios in real-life routine clinical practice. Mediation refill rates were similar in the two arms as all patients fulfilled their prescriptions at the hospital pharmacy on the same day of the clinical encounters. Finally, we did not measure patients’ behaviour change at home and patients may make lifestyle adjustment, such as reducing salt intake. However, this should be equally possible in both intervention and control groups given patients blinded in the trial.

In conclusion, a treatment strategy guided by haemodynamic measurements reduced BP more effectively than standard care in this trial in China. These findings justify further large-scale studies to provide more definitive evidence.

## Data Availability

Data are available upon reasonable request. Technical appendix, statistical code and dataset are available upon request from the corresponding author.
