# Correction: *Effectiveness of an impedance cardiography guided treatment strategy to improve blood pressure control in a real-world setting: results from a pragmatic clinical trial*

**DOI:** 10.1136/openhrt-2021-001719corr1

**Published:** 2021-10-28

Lu Y, Wang L, Wang H, *et al*. Effectiveness of an impedance cardiography guided treatment strategy to improve blood pressure control in a real-world setting: results from a pragmatic clinical trial. *Open Heart* 2021;8:e001719. doi:10.1136/openhrt-2021-001719

This article has been corrected since it was first published. The order of authors has been amended so that Dr. Luyan Wang is listed first and Dr. Yuan Lu is listed second.

