# Supplementary material for: Effectiveness of an impedance cardiography guided treatment strategy to improve blood pressure control in a real-world setting: results from a pragmatic clinical trial
Source: Open Heart. 2021 Sep 27;8(2):e001719. doi: 10.1136/openhrt-2021-001719 (PMC8477318; doi:10.1136/openhrt-2021-001719)
Supplement: Supplementary data [file openhrt-2021-001719supp001.pdf]

Effectiveness of An Impedance Cardiography Guided Treatment Strategy to Improve Blood Pressure Control in A Real-World Setting: Results from A Pragmatic Clinical Trial

Online Supplement

Table of Contents

Content	First Page
**Appendix Text 1.** Original Study Protocol	1
**Appendix Text 2.** Description of database of 114,198 generally healthy Chinese adults	6
**Supplemental Figure S1.** Enrolment and randomization of study participants	7
**Supplemental Table S1.** Baseline hemodynamic profiles of study participants by hemodynamic phenotype and intervention group	8
**Supplemental Table S2.** Baseline characteristics of study participants by hemodynamic phenotype and intervention group	9
**Supplemental Table S3.** Number and class of medications prescribed at baseline and follow-up visit in hemodynamic and standard care groups	10
**Supplemental Table S4.** BP reduction and achievement of BP goals in hemodynamic and standard care groups	11

Wang L, *et al. Open Heart* 2021; 8:e001719. doi: 10.1136/openhrt-2021-001719

Appendix Text 1. Original Study Protocol

I. Background

Patients with hypertension have been treated according to hypertension guidelines and physicians' experience, but several studies show that only about 35% with hypertension have their blood pressure under control. One of the reasons is that patients have diverged conditions of disease and risk factors, and the selection of a medicine and dosage regimen is sometimes suboptimal, leading to poor patient outcomes. Therefore, rational prescribing taking into account the variation in the patients is beneficial to improve the blood pressure control rate.

In this study, a non-invasive hemodynamic monitoring device: Impedance Cardiography (ICG) is used to measure the hemodynamic parameters of hypertensive patients, which can distinguish hyperdynamic, high-resistance, high-volume or hybrid phenotypes of hypertension. The goal is to optimize the treatment regimen according to the patient's hemodynamic characteristics. We plan to conduct a single-center, pragmatic randomized trial involving adults with uncontrolled hypertension who seek outpatient care at the Peking University People's Hospital between June and December 2019 in Beijing, China. This will improve the control rate and reduce medical costs. This study explores the correlation between the efficacy of common antihypertensive drugs and the hemodynamic characteristics of patients, uses a simple ICG technique to distinguish the hemodynamic phenotypic characteristics of the hypertensive population, and explores the clinical value of the treatment of hypertensive patients based on the hemodynamic phenotypic characteristics of hypertension.

II. Study objectives

1. Understand the hemodynamic characteristics of hypertensive patients.
2. Compare the control rate of blood pressure with and without hemodynamic information, including the changes of blood pressure and control rate

III. Efficacy parameters

Primary parameters: the change from baseline in SBP and DBP in 2 months post baseline
Secondary parameters: the BP control rate at 2 months post baseline

IV. Inclusion criteria

1. Inclusion criteria.
1) meeting the diagnostic criteria for hypertension of the Chinese Guidelines for the Prevention and Treatment of Hypertension 2018, with current guideline blood pressure ≥ 140/90 mmHg.
2) Patients with primary hypertension, previously untreated or treated with 1-2 antihypertensive

drugs

3) Age 18 to 85 years

4) Agree to sign the informed consent form

2. Exclusion criteria.

1) Unstable hemodynamic disease, or acute myocardial infarction, heart failure within 6 months, and chronic kidney disease CKD < 45ml-min-1-(1.73m2) -

2) High doses of diuretics as well as B-blockers (usually double doses), which cannot be stopped

3) Atrial fibrillation with severe arrhythmias.

4) Severe aortic regurgitation

5) Pleural effusion

6) Height and weight out of range: 120-230 cm, 30-230 kg

7) Use of 3 or more hypertensive drugs

8) Known secondary hypertension.

9) Refusal to sign the informed consent form.

V. Study methods

1. Method of measurements

1) blood pressure measurement equipment: Omron automatic electronic blood pressure measurement device model: HBP1300 (certified by the UK, US and EU).

2) Cardiac hemodynamic monitor (ICG) (software configuration: Hemodynamics™, model CHM P2505, certified by China's National Medicinal Products Agency)

2. Study design

1) All patients take blood pressure measurements at seated and supine positions. Seated blood pressure measurement: outpatient clinic office, 30 minutes of sitting rest, no smoking, alcohol or coffee before blood pressure measurement. Three consecutive blood pressure measurements were taken with the sitting arm parallel to the atrial level, with an interval of 1 minute each time, and the difference in blood pressure values should be less than 4 mmHg for each measurement, and the average value was taken.

2) All patients take cardiac hemodynamic measurements (ICG) at baseline visit. Sitting and lying blood pressure measurements are taken before ICG measurements, with the same method as above in the sitting position and 5 minutes in the lying position, with the blood pressure measurement arm abducted at 45 degrees, and the same measurement time and method as above.

3) Record the available hypertension treatment drugs and the dose used

4) Observe the attainment status and hemodynamic characteristics of blood pressure in hypertensive patients at baseline level and after two months.

5) Patients are randomly divided into two groups (hemodynamic-guided group, conventional treatment group) in a 1:1 ratio, with stratified randomization factors including the study center and the number of medications currently taken at baseline visit.

Randomization procedure: At the baseline visit, the system automatically generates randomized groups to assign patients to the hemodynamic or conventional treatment groups. At the 2-month visits, the subject grouping remains the same treatment group. The nurses who take the BP and ICG measurements are blinded to the treatment assignment.

ICG Report forms: physicians are given the ICG report forms if the patients are randomly assigned to the hemodynamic group, while physicians are not given the ICG report forms if the patients are randomly assigned to the conventional treatment group

VI. Efficacy parameters

1) Change from baseline in SBP and DBP at 2-month post baseline
2) BP control rate at 2-month post baseline
3) Change from baseline in hemodynamic parameters: CI, HR, AS, SVRI, TBR

VII. Statistical analysis methods

The total sample size of this study is to be 100 individuals (50:50) . This sample size is considered to be adequate to detect desirable differences between the treatment groups.

Statistical analyses will take into account the various influencing factors. p-values less than 0.05 are considered statistically significant.

The statistical approach will be based on ANCOVA model, and the model dependent variables include: systolic blood pressure, diastolic blood pressure, mean arterial pressure, and hemodynamic parameters (CI, AS, SVRI, TBR, etc.) ; independent variables include: subgroup factors, central factors, and individualized factors such as baseline blood pressure, baseline hemodynamic parameter values, baseline antihypertensive medication, age, gender, height, and weight. Subgroup analysis will consider age, gender, treated/untreated, baseline hemodynamic phenotype classification, and baseline hypertension medication.

VIII. Safety assessment

This study does not involve safety assessment.

IX. Study management

Informed Consent

1) The investigator or his representative will explain to the subject the nature of the study and answer all questions regarding the study.

2) Subjects must be informed of their voluntary participation in the study.

3) Subjects must sign an informed consent form that meets the requirements of the regulations, ethics committee or research center. The person authorized to obtain informed consent must also sign the informed consent form.

4) A copy of the informed consent form must be provided to the subject or the subject's legal representative.

5) The original signed and dated informed consent form will be retained at the Research Center and must be kept in a secure location.

Data Protection

1) After informed consent has been obtained, a unique subject number will be assigned to patients enrolled in the study. All subject records or data sets forwarded to the sponsor will contain only the subject number; the subject's name or any information that identifies him/her will not be forwarded.

2) Subjects must be informed that the study will use their personal data in accordance with laws and regulations relating to data protection and privacy protection. The extent of the data disclosure must also be explained to the subject.

3) Subjects must be informed that their medical records may be accessed by other authorized personnel designated by the sponsor, members of relevant ethics committees, and inspectors from regulatory agencies. All such persons will treat the subject's information in strict confidence.

Study Management

In accordance with the Code of Quality Management for Medical Device Clinical Trials, the Principal Investigator makes decisions and discussions on behalf of all investigators of this study.

This study will be posted on the following clinical study registry website: clinicaltrials.gov

Regulatory and Ethical Considerations

The study will be conducted in accordance with the study protocol and the following requirements.

- Declaration of Helsinki
- The Code of Practice for the Quality Management of Clinical Trials of Medical Devices
- Applicable laws and regulations

The protocol, protocol amendments (if applicable) , informed consent, and other relevant documents must be submitted to the Ethics Committee by the investigator and reviewed and approved by the Ethics Committee prior to the start of the study.

Data Quality Assurance

1) All subject data will be recorded in a paper CRF, and the investigator is responsible for manually signing the CRF as a means of confirming the completeness, accuracy, legibility, and timeliness of the data.

2) The investigator is required to maintain the original data to support the information in the CRF.

3) The investigator must allow for study-related monitoring, ethics committee review, regulatory

agency visits, and direct access to study documents and raw data.

Appendix Text 2. Description of database of 114,198 generally healthy Chinese adults

For each patient, the clinical decision support system determined his or her hemodynamic phenotype using personalized cutoffs of hemodynamic parameters (e.g., HR, CI, AS, SVRI, TBR) based on patient's gender, age, weight, height and BMI, using data from a large sample of 114,198 generally healthy Chinese adults. This database have been previously described.[1,2] In brief, a total of 116,851 individuals (65,172 men and 51,679 women), aged 20-80 years, underwent impedance cardiography (ICG) — a non-invasive method of measuring the hemodynamic parameters — at 51 sites of iKang Health Checkup Centers throughout China between January 2012 and October 2018. The ICG test was offered to all individuals who came in for a routine annual physical examination at these health checkup centers as a part of their employment benefits, and the majority were urban, working class, asymptomatic patients. We excluded 2653 people with outliers of weight, height, blood pressure (BP), heart rate, stroke volume, and baseline thoracic impedance due to concerns regarding accurate measurement of ICG parameters in these individuals. The remaining 114,198 individuals (64,229 men and 49,969 women) with accurate ICG measurements were used to determine the personalized cutoffs of hemodynamic parameters. Information on the individual's age, sex, geographical region, weight, height, SBP, and DBP was collected by nurses at the health checkup centers. Weight was measured using a standardized instrument to the nearest 0.1kg and height was measured to the nearest 0.1 cm using a portable stadiometer (Omron HNJ-318; Omron Corporation, Kyoto, Japan) with patients standing without shoes and heels against the wall. Body mass index (BMI) was calculated as a person's weight in kilograms divided by the square of height in meters. BP was recorded, after resting for 5 mins in a seated position, on the right arm using an automated blood pressure monitor (Omron HBP-9020; Omron Corporation, Kyoto, Japan) soon after which the patients underwent the ICG test. This standardized protocol was followed consistently across all sites.

Reference:

1. Mahajan S, Gu J, Lu Y, et al. Hemodynamic Phenotypes of Hypertension Based on Cardiac Output and Systemic Vascular Resistance. Am J Med 2020;133:e127-e39.
2. Mahajan S, Gu J, Caraballo C, et al. Relationship of Age With the Hemodynamic Parameters in Individuals With Elevated Blood Pressure. J Am Geriatr Soc 2020.

Wang L, *et al. Open Heart* 2021; 8:e001719. doi: 10.1136/openhrt-2021-001719

Supplemental Figure S1. Enrolment and randomization of study participants

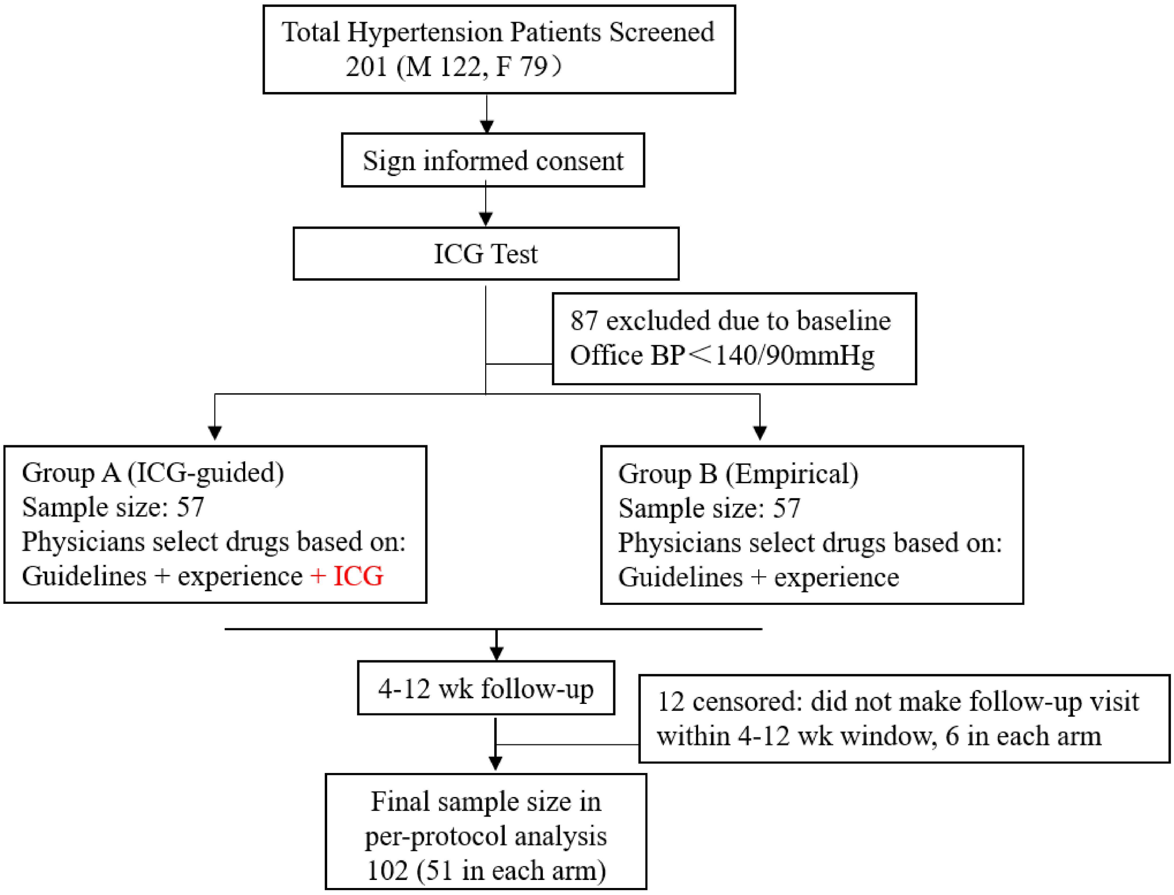

Supplemental Table S1. Baseline hemodynamic profiles of study participants by hemodynamic phenotype and intervention group

	Intervention Group				Control Group			
	Cardiac phenotype (high HR or high CI)	Arterial vascular phenotype (high AS)	Peripheral vascular phenotype (high SVRI)	Volemic phenotype (high TBR)	Cardiac phenotype (high HR or high CI)	Arterial vascular phenotype (high AS)	Peripheral vascular phenotype (high SVRI)	Volemic phenotype (high TBR)
N	13	11	30	17	13	18	26	11
HR (BPM)	81 (11.7)	75 (8.7)	71 (11.1)	68 (11.1)	77 (10.8)	72 (9.2)	69 (9.6)	68 (10.8)
CI (L/min/m^2)	3.65 (0.69)	2.85 (0.48)	2.75 (0.43)	3.12 (0.70)	3.71 (0.74)	2.86 (0.49)	2.7 (0.55)	2.91 (0.68)
AS (mmHg/mL/b)	0.78 (0.35)	1.01 (0.34)	0.83 (0.35)	0.78 (0.47)	0.72 (0.25)	0.97 (0.25)	0.82 (0.29)	0.79 (0.39)
SVRI (dyn·s·m2/cm5)	2496(649)	3249 (763)	3400(467)	2999 (722)	2404 (536)	3135 (679)	3411 (425)	3085 (690)
TBR (%)	0.8 (0.11)	0.76 (0.11)	0.78 (0.12)	0.88 (0.09)	0.79 (0.11)	0.77 (0.12)	0.74 (0.10)	0.87 (0.07)

Wang L, *et al. Open Heart* 2021; 8:e001719. doi: 10.1136/openhrt-2021-001719

Supplemental Table S2. Baseline characteristics of study participants by hemodynamic phenotype and intervention group

	Intervention Group				Control Group			
	Cardiac phenotype (high HR or high CI)	Arterial vascular phenotype (high AS)	Peripheral vascular phenotype (high SVRI)	Volemic phenotype (high TBR)	Cardiac phenotype (high HR or high CI)	Arterial vascular phenotype (high AS)	Peripheral vascular phenotype (high SVRI)	Volemic phenotype (high TBR)
N	13	11	30	17	13	18	26	11
Male, n (%)	10 (77%)	6 (55%)	17 (57%)	12 (71%)	4 (31%)	12 (67%)	15 (58%)	10 (91%)
Female, n (%)	3 (23%)	5 (45%)	13 (43%)	5 (29%)	9 (69%)	6 (33%)	11 (42%)	1 (9%)
Age, mean (SD)	53.8 (14.0)	59.6 (7.8)	53.1 (11.4)	56.4 (13.3)	52.8 (13.8)	51.9 (16.0)	50.5 (13.9)	61.4 (13.2)
BMI (kg/m^2)	26.4 (5.2)	25.7 (4.0)	26.4 (4.4)	26.1 (3.3)	24.6 (3.2)	28.3 (4.7)	28.3 (4.4)	27.3 (2.4)
SBP (mmHg)	149.8(10.2)	156.4(13.6)	152.4(13.6)	149.9(11.7)	145.2(10.5)	156.2 (9.5)	152.2(11.3)	149.3(7.8)
DBP (mmHg)	88.6 (8.8)	90.3 (13.7)	96.3 (6.6)	91.8 (10.1)	89.2 (8.9)	88.6 (16.6)	94.2 (10.6)	87.1 (12.2)
Diabetes, n (%)	1 (7.7%)	2 (18.2%)	7 (23.3%)	5 (29.4%)	2 (15.4%)	2 (11.1%)	6 (23.1%)	3 (27.3%)
CHD, n (%)	0 (0%)	0 (0%)	2 (6.7%)	0 (0%)	0 (0%)	1 (5.6%)	1 (3.8%)	0 (0%)
Stoke, n (%)	0 (0%)	0 (0%)	0 (0%)	1 (5.9%)	0 (0%)	0 (0%)	0 (0%)	0 (0%)
CKD, n (%)	1 (7.7%)	1 (9.1%)	2 (6.7%)	4 (23.5%)	0 (0%)	0 (0%)	1 (3.8%)	1 (9.1%)

Supplemental Table S3. Number and class of medications prescribed at baseline and follow-up visit in hemodynamic and standard care groups

	Baseline			Follow-up		
	Intervention group (n=51)	Control group (n=51)	*P value*	Intervention group (n=51)	Control group (n=51)	*P value*
Antihypertensive medication (n±SD)	1.39±0.18	1.41±0.16	0.988	2.37±0.16	2.41±0.18	0.812
RASI	41%	45%	0.842	76%	84%	0.455
BB	39%	35%	0.838	63%	47%	0.163
CCB	27%	31%	0.828	47%	67%	0.071
DD	31%	29%	1.000	52%	35%	0.161

RASI: renin-angiotensin system inhibitors, BB: beta-blockers, CCB: calcium channel blockers, DD: diuretics, SD: standard deviation.

Supplemental Table S4. BP reduction and achievement of BP goals in hemodynamic and standard care groups

	N	Baseline BP (mmHg)		Follow-up BP (mmHg)		BP reduction (mmHg)		Goal achievement rate (%)
		SBP	DBP	SBP	DBP	SBP	DBP	
Intervention group	51	151.8 ± 12.6	92.7 ± 9.6	131.9 ± 10.9	81.4 ± 7.7	-19.9 ± 10.7***	-11.3 ± 6.2***	67**
Control group	51	150.0 ± 10.3	89.5 ± 12.6	138.0 ± 13.7	84.6 ± 12.9	-12.0 ± 11.8	-4.9 ± 9.9	41

SBP: systolic blood pressure, DBP: diastolic blood pressure, BP: blood pressure.

** p<0.05， ***p<0.001

Wang L, *et al. Open Heart* 2021; 8:e001719. doi: 10.1136/openhrt-2021-001719
